# Detection of Atrazine and Its Metabolites in Natural Water Samples Using Photonic Molecularly Imprinted Sensors

**DOI:** 10.3390/molecules27165075

**Published:** 2022-08-10

**Authors:** Zahra Salahshoor, Khanh-Van Ho, Shu-Yu Hsu, Adel H. Hossain, Kathleen Trauth, Chung-Ho Lin, Maria Fidalgo

**Affiliations:** 1 Department of Civil and Environmental Engineering, University of Missouri, Columbia, MO 65211, USA; 2 Center for Agroforestry, School of Natural Resources, University of Missouri, Columbia, MO 65211, USA; 3 Department of Chemistry, University of Missouri, Columbia, MO 65211, USA; 4 Molecular Imaging and Theranostics Center, University of Missouri, Columbia, MO 65211, USA; 5 Department of Food Technology, Can Tho University, Can Tho 92000, Vietnam

**Keywords:** molecularly imprinted polymers, pesticides, natural water, sensors

## Abstract

In a previous study, photonic-based molecularly imprinted polymers (MIPs) were fabricated using atrazine (ATZ) and its metabolites, desethylatrazine (DEA) and desisopropylatrazine (DIA), as templates in separate matrices. For the purposes of monitoring the abovementioned molecules in natural waters, the effect of natural waters—featuring ionic strength and natural organic matter (NOM) on atrazine MIP—were studied in this work, and the photonic MIP was implemented for monitoring the target molecules in natural water samples collected from land in nearby farms in northeast of Columbia MO. Non-imprinted polymers (NIP) were also fabricated and applied in the experiments as a control test. In presence of NaCl, CaCl_2_, and NOM, MIPs presented lower responses by 26%, higher responses by 23%, and higher responses by 35%, respectively. NIPs response in terms of an increase or decrease was consistent with those of MIPs, but only for a lower percentage. MIPs response in natural waters—which were characterized for their physicochemical characteristics such as conductivity, total organic carbon content, etc.—provided a good approximation of the real concentrations obtained from the LCMS instrument; in general, they showed a good concordance, although large discrepancies occurred for some samples, which can be related to reproducibility issues in the manufacturing process or the presence of unknown interfering compounds in the real samples.

## 1. Introduction

Wetlands are lands that transition between terrestrial and aquatic systems where the water table is usually at or near the surface, or the land is covered by shallow water or saturated with water, from groundwater flowing up from an aquifer, from a nearby lake or river, or created by strong tides of seawater that form coastal wetlands. Wetlands are known as the most diverse ecosystems; they improve water quality and nutrient cycling, conserve the composition of atmosphere, and decrease chances of flooding [1]; therefore, their protection is of utmost importance. Different threats, such as habitat loss and degradation, climate change, pollution, overharvesting and disease, and excessive application of pesticides and fertilizers, jeopardize wetlands [2]. Wetland water quality monitoring in rural areas is fundamental in order to recognize the extent of contamination and preserve wetland ecology and water health.

**Figure 1:**
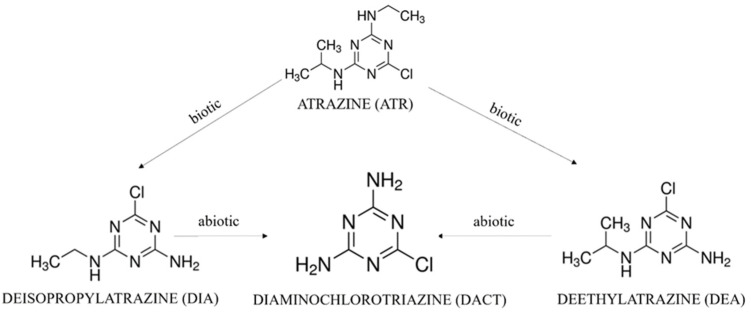
Structure of atrazine and its hydrophilic metabolites, DIA, HA, and DEA.

Atrazine is a triazine herbicide; this is most commonly applied in the United States by farmers against broadleaf weeds and annual grasses [3] and it has the potential to contaminate the water and soil. Due to its toxicity, the United Sates Environmental Protection Agency (USEPA) established a drinking water maximum contaminant level (MCL) for atrazine of 3 μg/L [4]. Chronic exposure to higher concentrations may cause lung, heart, and kidney diseases, low blood pressure, muscle spasms, weight loss, and damage to adrenal glands [5]. Atrazine is metabolized in the environment through biotic and abiotic pathways and some of those metabolites have also raised concerns due to their potential health effects [6,7] (Figure 1).

Although practices that minimize atrazine runoff and make it hold onto the field have been promoted [8], atrazine has been detected in the natural environment. Water soluble metabolites, deisopropylatrazine (DIA; 6-chloro-*N*-ethyl-[1,3,5]triazine-2,4-diamine) and deethylatrazine (DEA; 6-chloro-*N*-isopropyl-[1,3,5]triazine-2,4-diamine), can be found along with the parent compound dissolved in natural waters and can be transferred through aquatic media.

Currently available analytical methods for the detection of these contaminants at trace levels include liquid or gas chromatography coupled with mass spectrophotometry (LC or GC/MS) and enzyme-linked immunosorbent assay (ELISA) [3]. Although they are relatively precise and specific for the selected analytes, they require extensive sample preparation in the laboratory by a skilled operator at adequate facilities, which makes them time consuming, expensive, and not suitable for detection in the field. We have previously reported the fabrication of a photonic molecularly imprinted (MIP) sensor for atrazine and two of its metabolites, desethylatrazine (DEA) and desisopropylatrazine (DIA), in the range of 0.1 μg/L to 10 μg/L [9]. Photonic MIPs are suitable for in situ applications; they are composed of a hydrogel film supported on a polymethylmethacrylate (PMMA) slide and they provide facile measurements expressed as a change in the wavelength of the light reflected by the film after exposure to the sample. The low concentrations expected and the complex chemical composition of natural waters make detection a challenging task. Sensing performance of the MIPs can be compromised by sample properties such as ionic strength and presence of natural organic matter. MIPs can be induced by changes in temperature [10], pH [11], and ionic strength [12]. For example, basic environments increase the response of MIPs due to changes in polymer charge and consequent swelling of the hydrogel, but extensive rinsing of the film before reading minimized the effect [13]. Other parameters that potentially can compromise performance of the MIPs is the presence of chemically similar compounds that are capable of obstructing the bonding of the intended target and blocking access to those sites, resulting in pore blockage, steric repulsion or electrostatic interactions at or near the surface of the film.

In this work, the performance of the fabricated MIP sensor was tested in natural water samples obtained from seven sampling sites nearby Columbia, Missouri. Sampling campaigns were conducted to investigate the levels of atrazine and its metabolites during preplant and cultivation seasons after rainfalls. Water samples were collected after precipitation events during the year and analyzed for concentrations of ATZ, DEA, and DIA, Total Organic Carbon (TOC) pH, temperature, and conductivity. Pesticide levels measured by the sensor on the original samples were compared to LC/MS values.

## 2. Materials and Methods

The following chemicals were purchased from Sigma-Aldrich (St. Louis, MO, USA) and used without any purification: acrylic acid (AA) (99%), ethylene glycol dimethacrylate (EGDMA) (98%), 2,2′-azobisisobutyronitrile (AIBN) (98%), hydrofluoric acid (HF) (48%), ethanol (99.5%, 200 proof), and acetic acid (96%). Atrazine was purchased from Santa Cruz Biotechnology (Dallas, TX, USA). Commercial silica particles (300 nm diameter) were supplied by Pinfire Gems and Colloids (Frankfurt, Germany). Suwannee River Natural Organic Matter (SRNOM) was purchased from International Humic Substances Society (St. Paul, MN, USA). Ionic strength of solutions was adjusted by NaCl (reagent grade, Acros Organics) and CaCl_2_ (reagent grade, J. T. Baker). Glass microslides (3″ × 1″ × 0.04″) were purchased from FisherBrand (Pittsburgh, PA, USA) and cut in 0.04″ × 1/3″ × 3″ pieces before use. Poly (methyl methacrylate) (PMMA) plastic slides of dimensions 0.04″ × 1/3″ × 3″ were obtained from ePlastics (San Diego, CA, USA).

### 2.1. Sensor Fabrication

**Figure 2:**
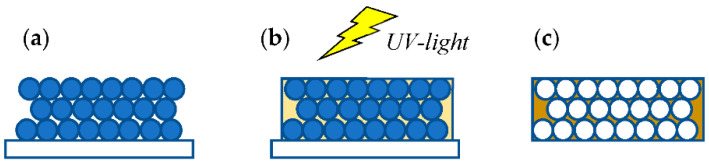
Schematic of MIPs fabrication: (**a**) colloidal crystal on a glass substrate; (**b**) infiltration with pre-polymerization solution and UV-polymerization; (**c**) porous film after etching of sacrificial particles and substrate by acid and target elution.

The steps for the fabrication of MIPs have been described elsewhere [9] and are schematically depicted in Figure 2. Briefly, a colloidal crystal was created by the self-assembly of SiO_2_ particles on a glass substrate; a pre-polymerization solution containing the functional monomer AA, the crosslinker EGDMA, the polymerization reaction initiator AIBN, the target molecule (ATZ, DEA, or DIA) and a solvent infiltrated the empty spaces of the colloidal crystal and was polymerized in situ under UV light. The substrate and SiO_2_ particles were etched away in a HF bath and, finally, the target eluted with acetic acid in an ethanol solution (volume ratio 1:9). Non-imprinted polymeric films (NIP) were fabricated as controls, following the same general procedure but in the absence of a target.

Scanning electron microscopy confirmed the colloidal crystal thickness between 12 and 15 layers, as well as the surface porosity and empty spherical cavities in the film given by the etched silica particles [14]. The NIP and MIP after target removal displayed similar chemical characteristics evidenced by the FTIR spectra, while MIPs before target removal showed the absorption band due to the interaction of the carboxylic and hydrogen atoms of amino groups or nitrogen atoms of triazine [9].

### 2.2. Natural Water Samples

**Figure 3:**
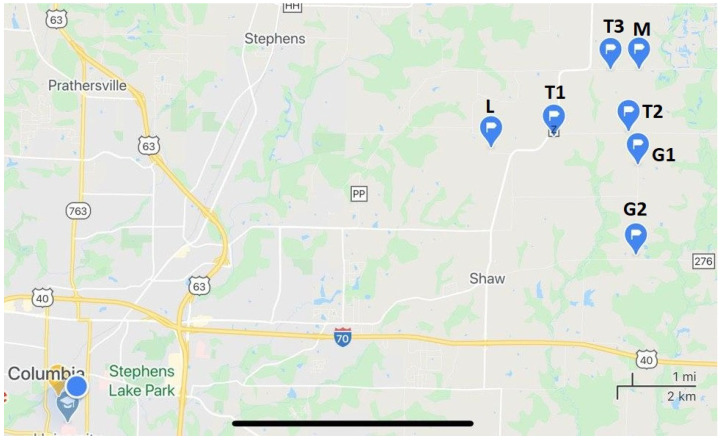
Sampling locations in the proximity of Columbia, Missouri.

Natural water samples were collected from 7 different locations northeast of Columbia, Missouri, as indicated in Figure 3: two mile Prairie School Rd 1 and 2 (T1 and T2), Judy School Rd (T3), Glendale Rd 1 (G1), Glendale Rd 2 (G2), Maupin Rd (M), and Liddel Ln (L). The sites are located at roadsides with public access and were chosen due to the potential for pesticide-containing runoffs due to their vicinity to the farms. Sampling was conducted in 2020 and 2021 during spring, summer, and fall immediately after precipitation events. In spring, summer, and fall, the samples were taken to analyze the effect of atrazine application and the amount detected in the runoffs in each period. An additional sampling took place at the end of the winter, before any atrazine application was expected.

A 10-L polyethylene bucket was used to receive at least 1 L of water. Immediately after sampling, pH and temperature were measured using a portable Oakton pHTester 30 (Vernon Hills, IL, USA). In some cases, due to an insufficient amount of rainfall or low humidity of soil, there was not enough water to obtain the minimum volume; then, the site was skipped for that occasion. Afterwards, water samples were transferred into 1000 mL propylene bottles (Waltham, MA, USA), labeled, and capped for storage in the lab at −18 °C until analysis. Photos were taken from each site to have a record of general conditions of the wetland such as vegetation, amount of water in each site, etc. Weather data from the Accuweather website were also saved to relate results from rain events and the analysis of samples.

### 2.3. Analytical Methods

Natural water samples were taken to the laboratory and analyzed for conductivity using a portable Fisher Scientific Traceable dual-display bench digital conductivity meter. The dissolved organic carbon concentration was measured using a Shimadzu TOC-VCPN analyzer.

The concentrations of atrazine (ATR), DEA, and DIA were determined by a Waters Alliance 2695 High Performance Liquid Chromatography (HPLC) system coupled with Waters Acquity TQ triple quadrupole mass spectrometer (MS/MS). The analytes were separated by a Phenomenex (Torrance, CA, USA) Kinetex C18 (100 mm × 4.6 mm; 2.6 µm particle size) reverse-phase column. The mobile phase consisted of 10 mM ammonium acetate and 0.1% formic acid in water (A) and 100% acetonitrile (B). The gradient conditions were 0–0.5 min, 2% B; 0.5–7 min, 2–80% B; 7.0–9.0 min, 80–98% B; 9.0–10.0 min, 2% B; 10.0–15.0 min, 2% B at a flow rate of 0.5 mL/min. The ion source in the MS/MS system was electrospray ionization (EI) operated in the positive ion mode with a capillary voltage of 1.5 kV. The ionization sources were programmed at 150 °C and the desolvation temperature was programmed at 450 °C. The MS/MS system was in the multi-reaction monitoring (MRM) mode with the optimized collision energy. The ionization energy, MRM transition ions (precursor and product ions; Table 1), capillary and cone voltage, desolvation gas flow, and collision energy were optimized by Waters IntelliStart™ optimization software package. The retention time, calibration equations, and limits of the detection for the analyses of ATR, DEA, and DIA are summarized in Table S1.

The concentration of atrazine and its metabolites in some samples were below LC-MS/MS limit of detection, and preconcentration by solid phase extraction (SPE) was required. For that purpose, atrazine (ATR), deethylatrazine (DEA), deisopropylatrazine (DIA), hydroxyatrazine (HA), and the internal standard terbuthylazine (TRB) in the water samples were extracted and concentrated by the SPE process. The water samples were filtered through a 0.2 μm Whatman Anotop syringe membrane filter (Sigma-Aldrich, St. Louis, MO, USA) and 50 mL of filtered samples were spiked with 500 µL of the internal standard terbuthylazine (TRB, 1 mg/L) to achieve a final concentration of 10 µg/L of TRB. Before the extraction, the Oasis HLB solid-phase extraction cartridges (500 mg; Waters, Milford, MA, USA) were conditioned with 8 mL of methanol, followed by additions of 8 mL of DI water to wash the cartridges twice. Following the condition and washing process, the samples (50 mL) were passed through the cartridges at a flow rate of 5 mL/min. After the samples were loaded, the cartridges were washed with 8 mL of DI water and sorbents were dried under vacuum in a SPE manifold system for 5 min. The analytes were subsequently eluted with 7 mL of methanol at 2 mL/min. The eluates were then concentrated under a stream of nitrogen in a temperature bath at 27 °C until dryness. The resulting extracts were resuspended with 1 mL of water: methanol (10:90, *v*/*v*), and then filtered through a 0.2 μm PTFE Acrodisc syringe membrane filter.

### 2.4. Pesticide Measurements Using MIP Photonic Sensor

The colloidal crystals used to obtain the film porosity yielded a 3D periodic porous structure which was responsible for the photonic properties of the sensor. The rebinding of the target molecules, when the clean sensor is exposed to a sample containing one of the pesticides, leads to a deformation (swelling) of the polymer, which translates in a readable optical signal using Bragg diffraction. The Bragg equation is defined as:

(1) $${ƛ}_{max\;}=1.633\left(\frac{d}{m}\right)\left(\frac{D}{D_{0}}\right)\sqrt{\left(n_{a}^{2}-\sin\theta^{2}\right)},$$

where *d* is the sphere diameter of the silica particle, *m* is the order of Bragg diffraction, (*D/D*_0_) is the degree of gel swelling (*D* and *D*_0_ are the diameters of the gel in the equilibrium state at a certain condition and in the reference state, respectively), *n_a_* is the average refractive index of the porous gel at a certain condition, and θ is the angle of incidence. Therefore, if the rebinding of target molecules causes any swelling or shrinkage in the hydrogel film, it is detected by optical signals.

A UV-Visible spectrophotometer (Cary 60, Varian, Palo Alto, CA, USA) with a Harrick Scientific’s Specular Reflection Accessory (ERA-30G) at a fixed angle of 30° was used in wavelength range of 200–800 nm and double-beam mode to measure the reflectance spectra of the polymeric films. In a typical experiment, the sensor was first immersed in DI water (blank) until equilibrium was reached, approximately 20 min [9]. Then, the sensor was taken out of the solution, pat dried softly and examined in UV-Vis spectrophotometer to record the reflectance spectrum. Secondly, the sensor was immersed in the sample solution for the same amount of time, and the process of acquiring the reflectance spectrum repeated. After inspection of both spectra, a shift in the peak wavelength can be calculated and correlated to the concentration of pesticide in the sample.

MIPs were tested in solutions with concentrations ranging from 0.1 μg/L to 10 μg/L (0.1 0.5, 1, 2.5, 5, and 10 μg/L) to generate a calibration curve for quantifying concentrations of the targets in unknown solutions. Pesticide concentrations in all laboratory prepared solutions were validated by LCMS/MS.

Natural waters exhibit a wide range of dissolved salts concentrations, from surface water, groundwater, or seawater. The effect of ionic strength on the sensor response was investigated with ATR solutions under different background conditions by the addition of NaCl or CaCl_2_, to yield 1 mM, 10mM, and 100 mM ionic strengths. Furthermore, plant waste, animal waste, or anthropogenic pollutions lead to organic matter content that is capable of adsorbing to the sensor surface and affect its response. In order to examine this effect, MIP and NIP responses were measured in solutions with different concentrations of ATR and 1 mg/L NOM.

## 3. Results

### 3.1. Natural Water Characterization and Pesticide Concentrations

The physicochemical characteristics of the natural water are presented in Table S2. The temperature of the samples changed as expected with the seasons and had similar values for all sites within each sampling date. The pH of the samples was neutral to slightly basic, ranging from 6.9 to 8.1. The conductivity of the samples changed from 33 to 197 μmhos/cm, reflecting variable amounts of dissolved salts present. Due to the differences in geotechnical properties of different sites and the amount of rain precipitated in each day, a dilution effect can be expected after the more important rain events, decreasing the conductivity of the sample.

TOC values ranged from non-detectable (<1 mg/L) to 10.93 mg/L. The dissolved oxygen concentration and level of stagnancy of the water in the streams affect the organic matter content in addition to the population of microorganisms available in each site, both contributing to different levels of TOC observed in the samples.

The concentration of ATR, DEA and DIA in the samples, as measured by LCMS/MS, is presented in Table 1. Concentrations of ATR and its metabolites are generally higher in samples taken on 10 June 2020, but they have also been detected in 12 and19 March 2020 samples, before the start of spring. Farmers apply part of atrazine to the crops at the end of winter, but most of it is applied in spring. As a consequence, higher levels of ATR and metabolites were observed in samples taken at the end of spring than in samples from the beginning of spring. Summer pesticide levels were the highest; in this time period, ATR is gradually washed off the farms with each rain event and emerges in surface water. The metabolite concentration also generally increases, as ATR molecules are partially degraded.

The data showed that on an average, sites T3, M, and T1 had the highest concentrations of ATR and its metabolites, and sites T2 and G1 are amongst the lowest analyte content. This could be due to a number of reasons, such as the sites distance from the point of atrazine application and circulation of runoffs after precipitation events. Data also showed that for most cases, ATZ was degraded to DEA more than DIA. However, the extent of its degradation to different metabolites in each site is not the same, hinting to the effect of different vegetations or microorganism population distribution in each site. For example, the images of T3 and L in Figure S1 clearly depict the difference in vegetation of two sites as well as the water amount, consequently impacting the biodegradation environment for the parent pesticide.

### 3.2. Effect of Ionic Strength with Different Salts

MIP equilibrium experiments were performed with atrazine solutions with different levels of background ionic strength (1, 10, and 100 mM), given by NaCl or CaCl_2_ to study the effect of the presence of charged ions on MIP response.

MIPs were first immersed for 20 min in atrazine-free water solutions of different ionic strengths (i.e., 1, 10, and 100 mM) containing each one of the electrolytes and the shift of the wavelength peak was recorded (Figure S2). In comparison with DI, the presence of NaCl produced a decrease in the swelling in the hydrogel and shrinkage of polymer film, resulting in lower peak wavelengths; incubation in CaCl_2_ solutions, on the other hand, led to increases in the peak wavelength of refection spectra, which hints to a higher swelling of the hydrogel. In both cases, the effect was minor, even at the relatively high IS levels, as the peak shifted less than 4 nm.

Figure S3 shows the response curve for MIPs incubated in atrazine solutions in DI water and aqueous solutions of NaCl at different concentrations. In the presence of NaCl, there was a decrease in peak wavelength shift observed, in accordance with previous experiments. As the ionic strength increased from 1 mM to 100 mM, the MIP response decreased by 26%.

It was reported that binding capacities of targets to MIPs were affected by cations following the Hofmeister series [15]. These experiments were carried out at near-neutral *p*H and due to carboxylic acid’s *p*K_a_ of approximately 5, most of the carboxylic groups in the polymer matrix were deprotonated. A higher concentration of NaCl resulted in higher charge densities at the surface of hydrogel, which suppressed the thickness of the electric double layer and caused less electrostatic repulsion and swelling of hydrogel compared to the conditions in DI water [13]. Thus, a shrunken hydrogel had less swelling and its reflectance spectrum had a lower peak wavelength, resulting in the underestimation of the target concentration in the presence of NaCl. The degree of underestimation augmented with solution ionic strength.

The measurement uncertainty, depicted by the error bars in Figure S3b, show that the response curve obtained under DI conditions can still be used as a calibration curve for solutions at 1 and 10 mM IS of NaCl, but not for higher ionic strengths, since the curve obtained in this case had large, overlapping error bars, and therefore, would not be considered accurate for measuring purposes.

In the presence of CaCl_2_ and as expected from the experiments in the absence of a target, the opposite result was observed. Increasing ionic strength from 1 mM to 100 mM using CaCl_2_ increased the peak wavelength shift by 23% (Figure S4). Comparing with the control in the absence of a target, the average wavelength shift decreased slightly at 1 mM IS for most atrazine concentrations; however, increasing the ionic strength, which corresponds to an increase in CaCl_2_ concentration, augmented the average peak wavelength shift and reverted the effect. A greater average shift is a result of a higher swelling ratio. It is reported that poly(acrylic acid) (PAA) is capable of high Ca^2+^ ion binding power [16]; Ca^2+^ binds to the carboxylate groups of PAA from neighboring polymer segments [17] due to the ion chelating property, hence, forming complexations with PAA. The incident of complexation resulted in swelling of the hydrogel and consequently, higher peak wavelengths occurred.

In the case of CaCl_2_, similar to NaCl, the response curve obtained under DI conditions is appropriate to be used as calibration to measure atrazine in solutions between 1 and 10 mM IS levels, since the intrinsic error of the MIPs is in the order of the difference in response for those solutions. Nevertheless, a difference in sensor responses in 100 mM ionic strong solutions are higher, and thus, the sensor cannot yield accurate results for those measurements if a specific high ionic strength calibration curve is not used. Additionally, results from incubating MIPs in solutions with different ionic strengths and absences of atrazine demonstrated that an increasing concentration of CaCl_2_ causes swelling of the hydrogel and yields larger peak wavelengths in reflectance spectra, which is in accordance with results from the incubation of MIPs in the presence of both ionic strength and atrazine.

### 3.3. Effect of NOM

In order to investigate the potential interference of NOM with the sensor response, MIPs were incubated in solutions with different concentrations of atrazine and a background concentration of 1 mg/L NOM. Control experiments were conducted with NIP films incubated in the same solutions and the results were compared with those in the absence of NOM.

The peak wavelength shift increased in the presence of NOM compared to the DI water atrazine solution (Figure S5). A larger peak wavelength shift for NIPs incubated in the presence of NOM than for those in DI water was generally observed, which was attributed to the absorbance of light by NOM, as some solutions may still remain after incubation within the porous film. When the experimental protocol was modified to include a thorough rinsing of the NIPs with DI water after taking them out from the solution in order to remove as much excess sample as possible from the surface and the internal porosity of the film before recording the spectra, the wavelength shift decreased to some extent, which further supports the hypothesis that some NOM molecules clung to the film, absorbed light, and caused the changes in peak wavelength observed in reflectance spectra. The same incident happened in the incubation of MIPs and measurement of their reflectance spectra, and the modified incubation protocol including the exhaustive rinsing of the sensor should be implemented in all cases when dissolved light absorbing molecules are suspected in the sample.

The comparison of the response curves in Figure S5 evidenced that the presence of 1 mg/L NOM in the solutions caused a 28% increase in peak wavelength shift of the NIPs and a 35% increase in the peak wavelength shift for the MIPs. This difference in the amount of shift change for MIPs and NIPs is due to the absence of sensor rinsing in the MIPs in these experiments. This amount of increase in the overestimation is within the intrinsic error of the MIPs and they can be used in natural waters with NOM of around 1 mg/L concentration.

### 3.4. MIPs Response in Natural Waters

**Figure 4:**
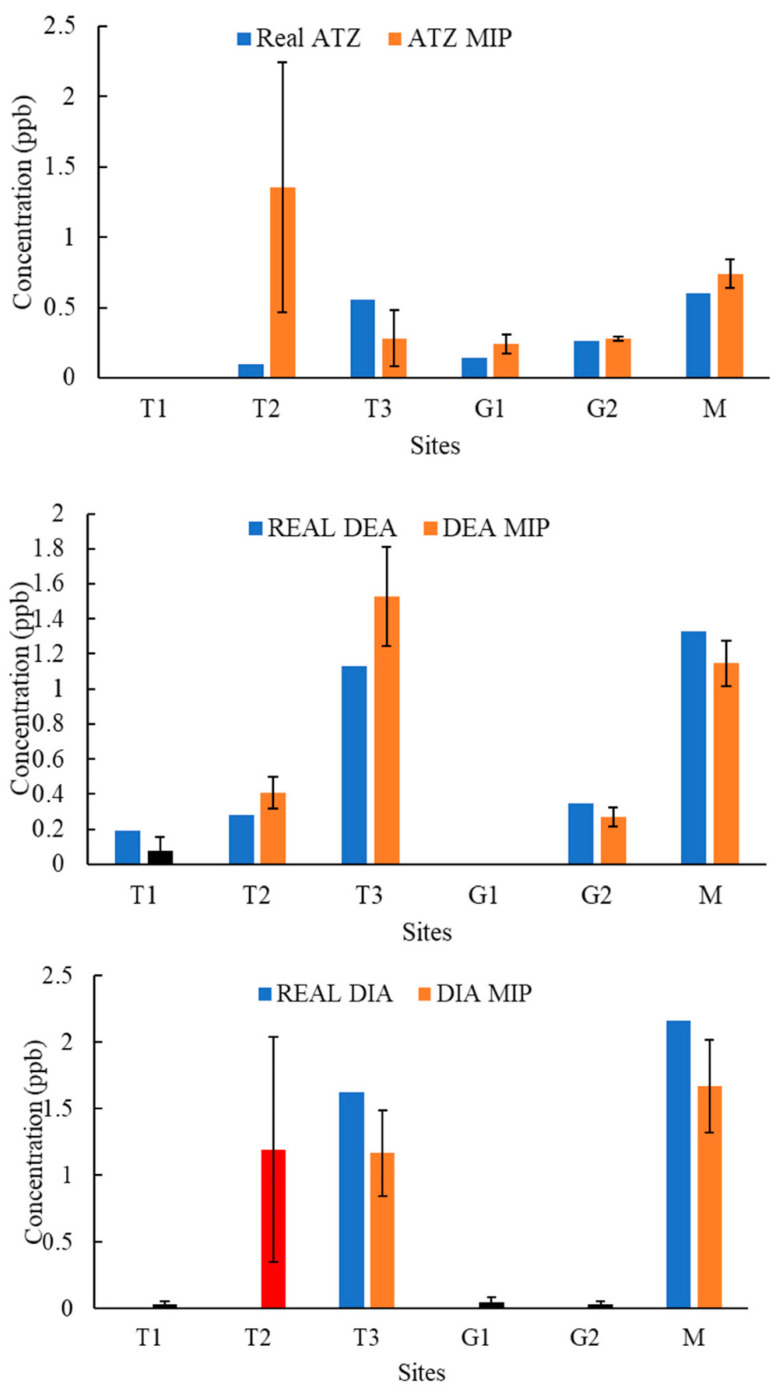
Average response of MIPs to ATZ, DEA, and DIA compared to real concentrations validated by LCMS/MS for samples of 12 March 2020.

**Figure 5:**
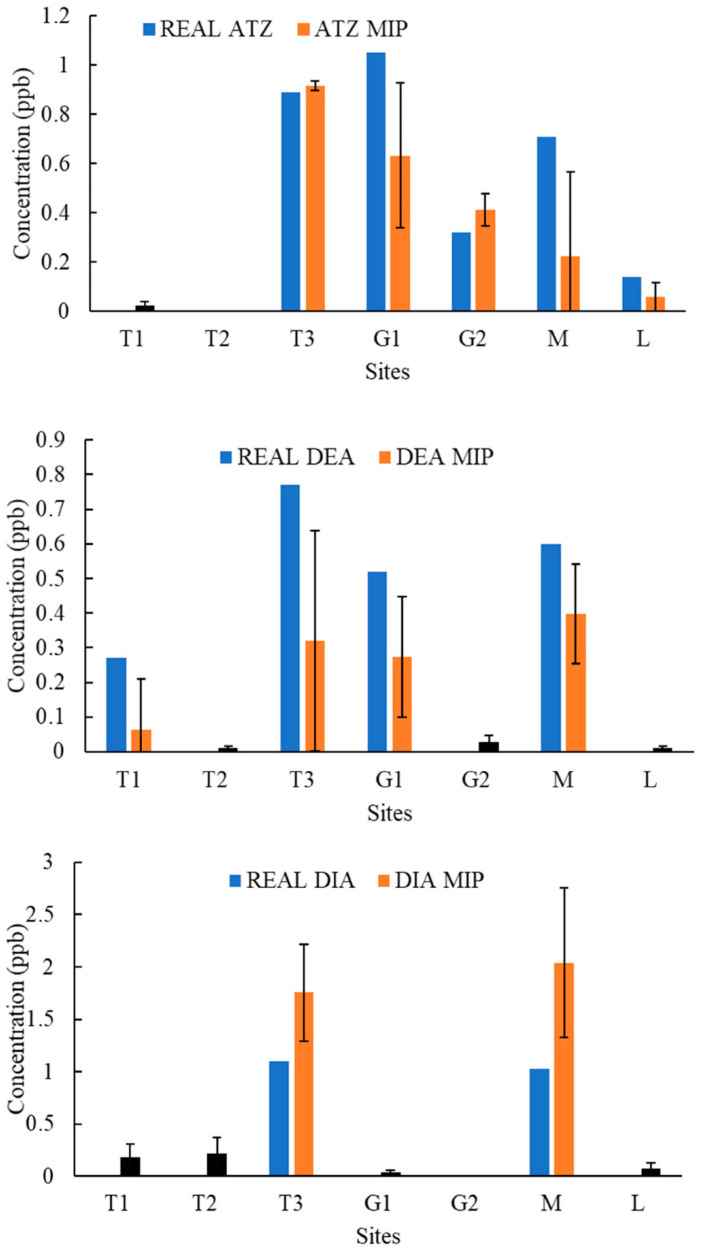
Average response of MIPs to ATZ, DEA, and DIA compared to real concentrations validated by LCMS/MS for samples of 19 March 2020.

**Figure 6:**
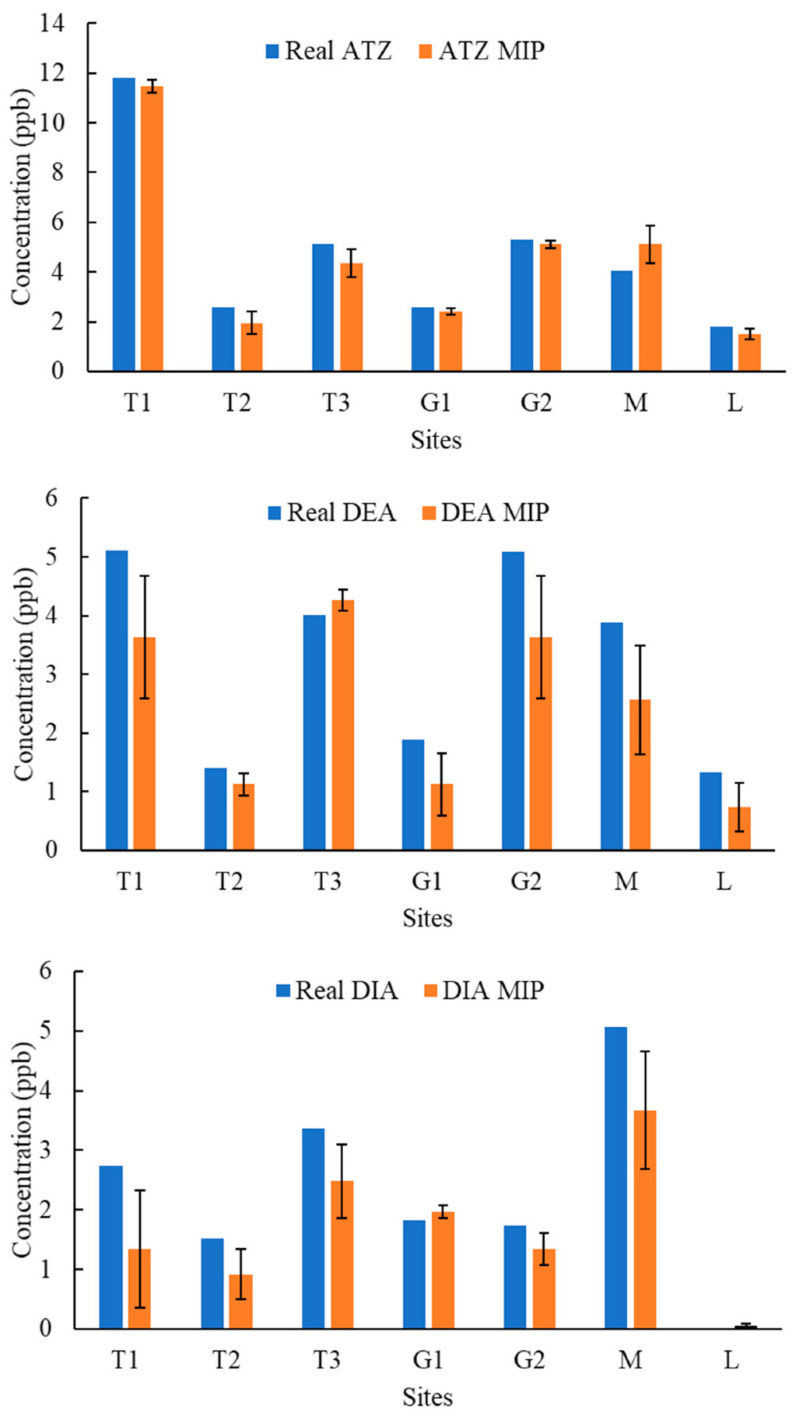
Average response of MIPs to ATZ, DEA, and DIA compared to real concentrations validated by LCMS/MS for samples of 10 June 2020.

**Figure 7:**
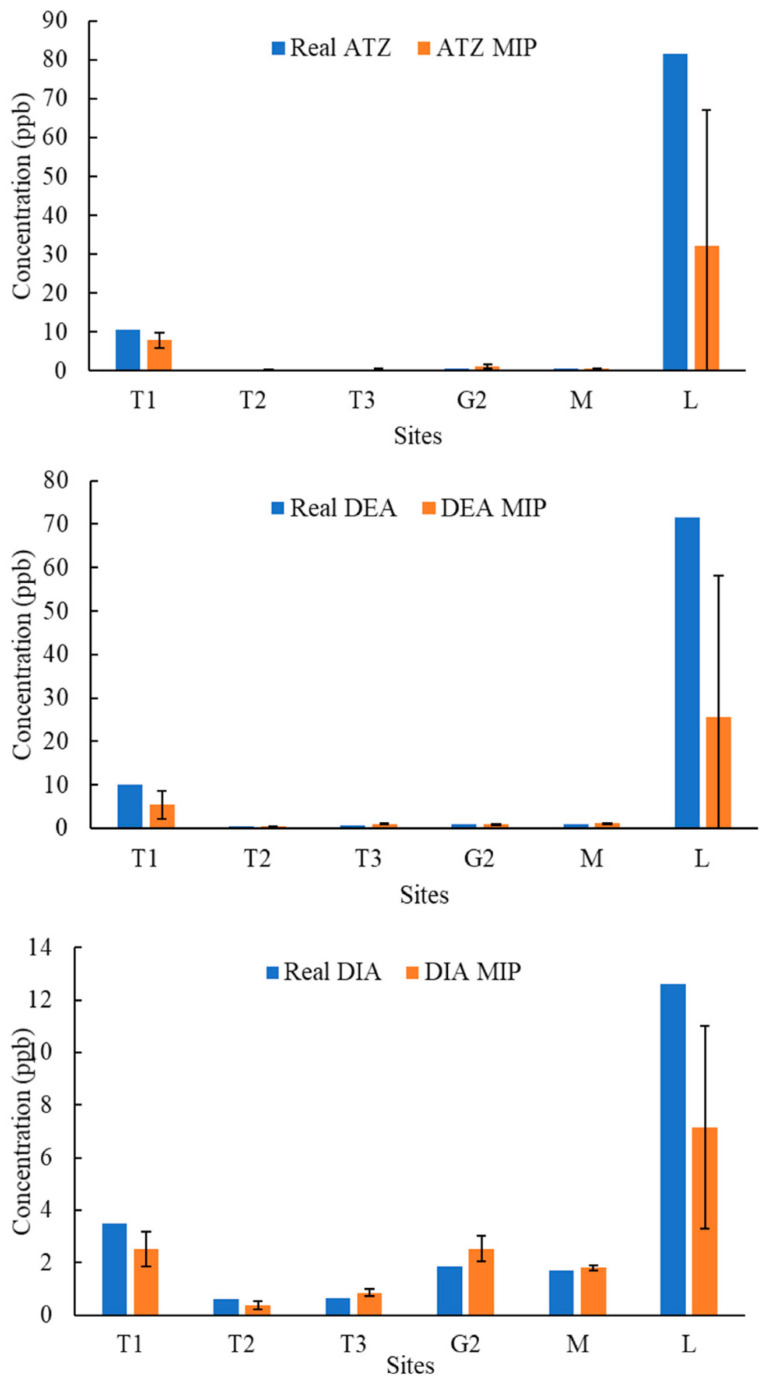
Average response of MIPs to ATZ, DEA, and DIA compared to real concentrations validated by LCMS/MS for samples of 11 August 2020.

**Figure 8:**
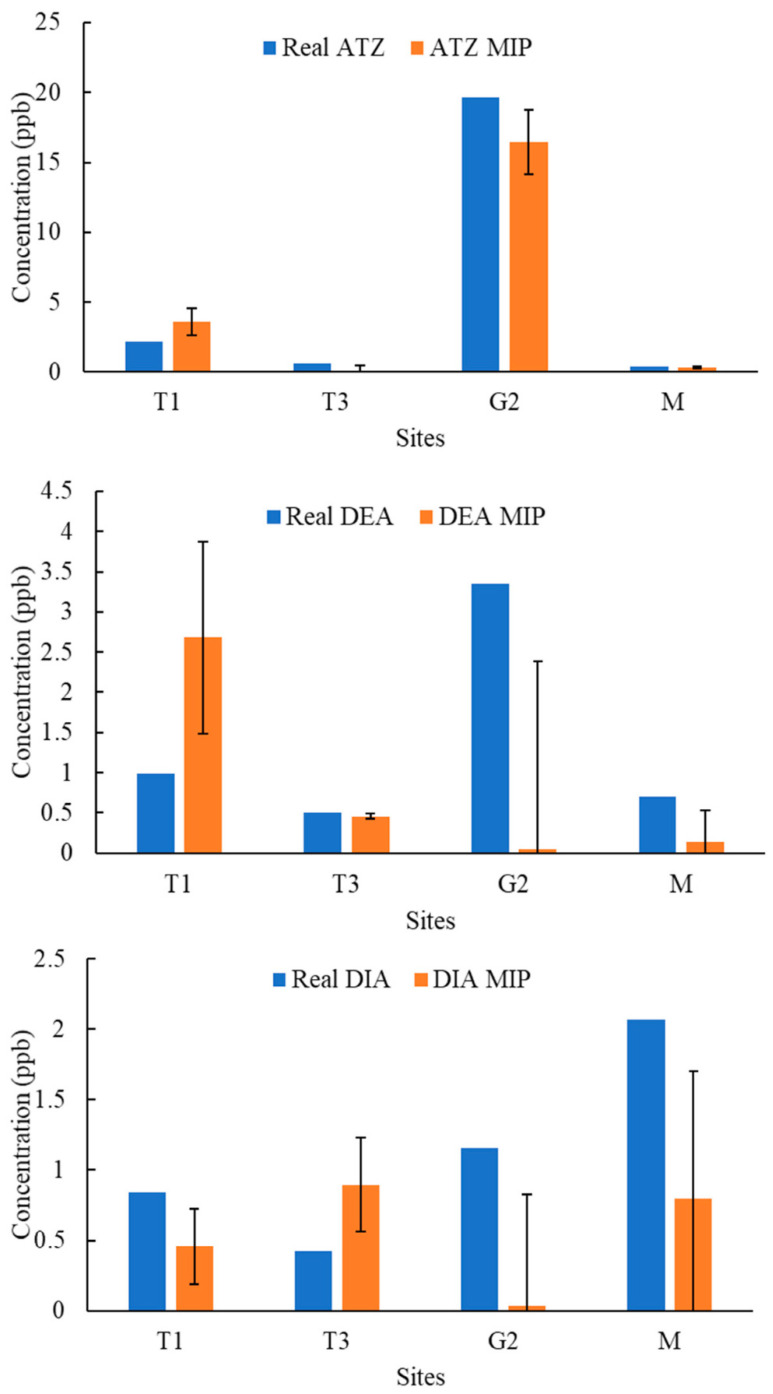
Average response of MIPs to ATZ, DEA, and DIA compared to real concentrations validated by LCMS/MS for samples of 23 October 2020.

**Figure 9:**
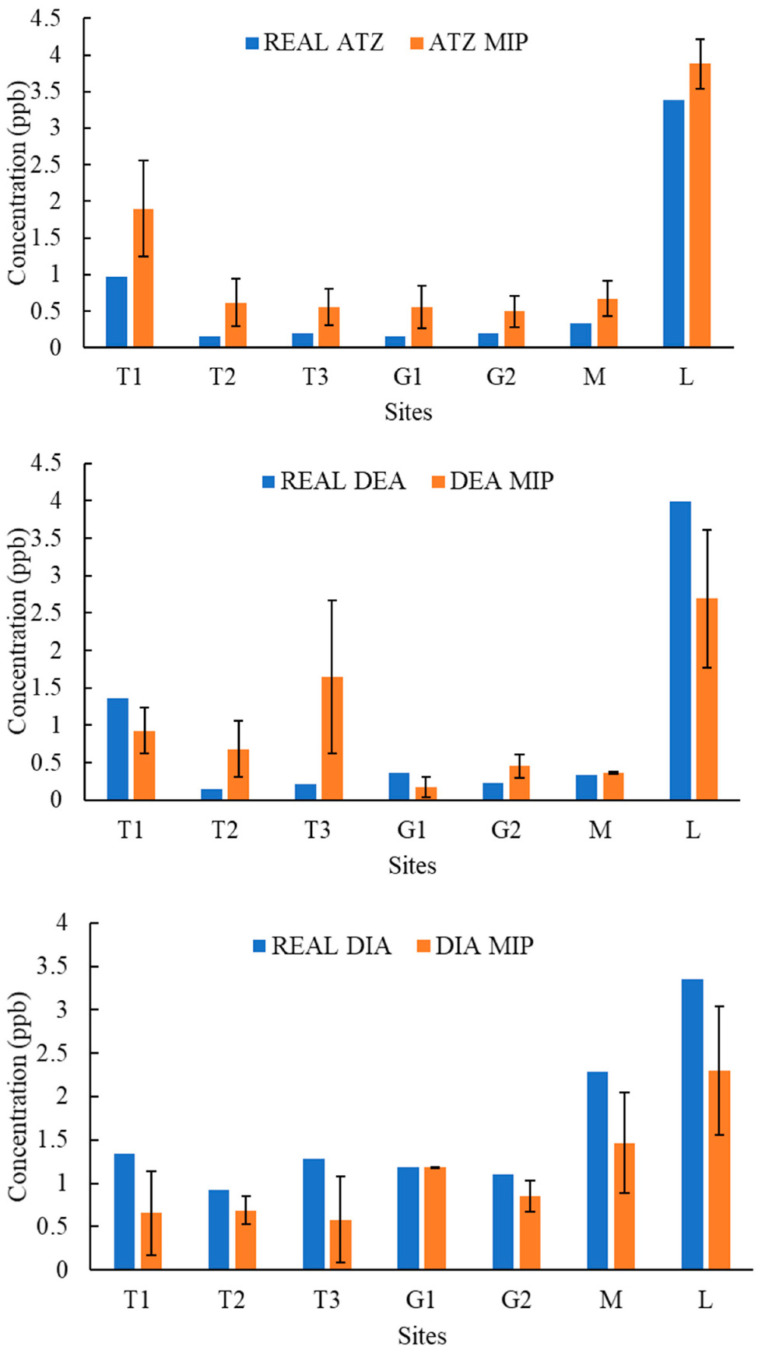
Average response of MIPs to ATZ, DEA, and DIA compared to real concentrations validated by LCMS/MS for samples of 14 March 2021.

**Figure 10:**
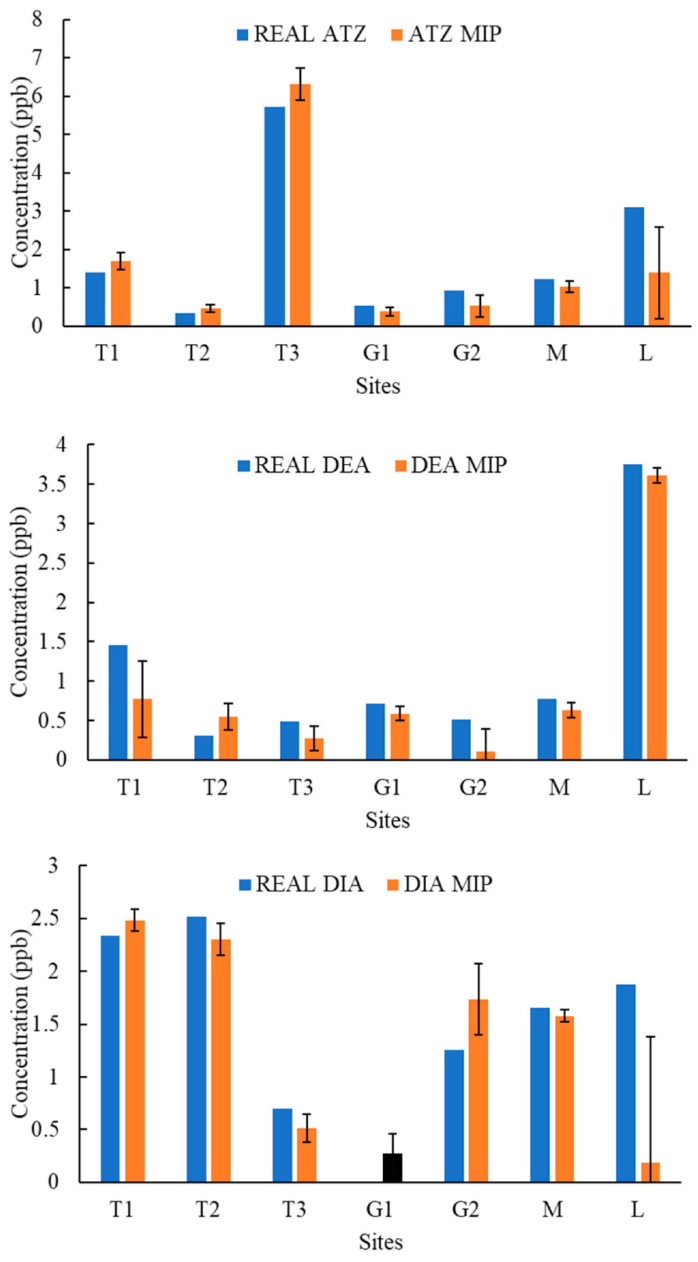
Average response of MIPs to ATZ, DEA, and DIA compared to real concentrations validated by LCMS/MS for samples of 24 April 2021.

Figure 4, Figure 5, Figure 6, Figure 7, Figure 8, Figure 9 and Figure 10 display the average response and analytical errors of MIP sensors and the concentrations of analytes measured by LCMS/MS as the true value for the seven sampling campaigns. Previous research had established LODs for the MIP-sensors to be 0.1, 0.2, and 0.3 μg/L for ATZ, DEA, and DIA MIPs, respectively [9]. Therefore, sensor measurements lower than LOD were represented in black, which correspond to a non-detect. The numerical values of the measurements are reported in Table S2, alongside the LCMS-MS determinations.

It was observed that the samples with highest conductivity, i.e., ionic strength, such as T2 of 12 March 2020 (Figure 4) and T2, T3, and M of 14 March 2021 (Figure 9), had a MIP response higher than a true concentration of the analytes. Depending on the ion being monovalent or divalent, MIP response could increase or decrease, the observe effect may be given by the presence of divalent ions in those samples given that they increase the conductivity, and PAA has a high affinity for their chelation [18]. In most cases for M and L sites, the MIP response is lower than a real concentration of the targets, which might suggest a higher content of monovalent ions in those sites or an absence of divalent species.

As demonstrated in the figures below, in most cases, and even for the sites with the highest content of TOC, the MIP response is relatively close or even lower than real concentration of the analytes, which was found to be in the range from 0 to 11 mg/L. However, the presence of natural organic matter is expected to produce an increase in the wavelength shift of the sensor. The careful rinsing of the sensor that was applied to these measurements would have reduced the overestimation of concentrations, and the present of monovalent ions may have partially offset the influence of NOM.

Chemicals other than the target compound for each MIP can potentially bind into the imprinted cavities if they have comparable molecular size and functional groups in common, and as a result, interfere in the accurate determination of the target. On the other hand, an increase in the number of molecules present in the solution may produce a detrimental effect in the diffusion of the target from the bulk of the solution to the binding sites, resulting in hindering or blocking the binding of the target, lower adsorption, and consequently, less pronounced peak wavelength shift [9]. Natural water samples are characterized by complex matrixes related to the particular environment as well as a myriad of compounds derived from anthropogenic activities that are largely unknown. It is very unlikely that the pesticides analyzed in this work were the only man-made substances in the water, and therefore, the interpretation of the MIP sensor errors remain highly speculative.

**Figure 11:**
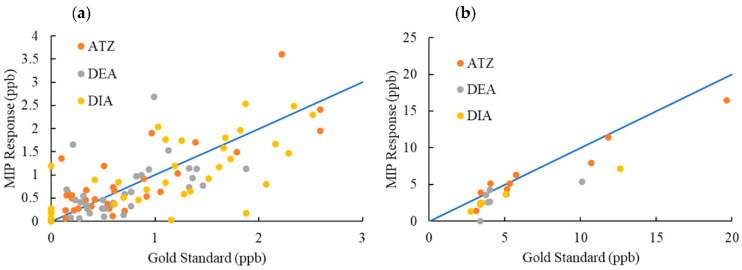
Degree of agreement (concordance) between MIP response and LCMS/MS measurements (gold standard); (**a**) 0 to 3 μg/L, (**b**) >3 μg/L.

The degree of concordance between the MIP sensor responses and the true value of analyte concentrations as measured by LCMS/MS widely considered the gold standard for analysis, as presented in Figure 11. Markers closer to the X = Y line indicate a better agreement of MIP response with the gold standard measurement. The data were divided into two different concentration regions: from 0 to 3 μg/L, and concentrations above 3 μg/L, to improve readability of the low concentration values. In most cases, MIP responses provided a good estimation of the true concentrations in relation to the results from the gold standard technique. Nevertheless, in few cases, the MIP sensor responses deviated from true values. As explained above, this discrepancy can be due to a number of reasons associated with the physicochemical characteristics of natural waters: ionic strength level, organic matter content, or other compounds present of similar molecular size or functional groups. Another source of error can be tracked down to the fabrication process of the sensor. The multiple steps in manufacturing of MIPs can introduce some variability in the morphology of the photonic films, and even minimal differences may decrease the reproducibility of the responses. This issue has recognized as a major challenge for the advancement of MIP-based technology to commercialization [19].

## 4. Conclusions

Photonic MIP sensors were applied to the determination of pesticide concentrations in natural waters collected from rural areas adjacent to farms after precipitation events during different seasons, and their performance was assessed by comparison to the analytical gold standard. The effect of ionic strength and NOM on the sensor response was investigated in the laboratory to receive insights into potential interfering conditions, showing that monovalent ions generally lead to the underestimation of concentrations (up to 26%), while divalent species with potential for specific chemical interactions produce higher than expected signals (23% for the highest concentration considered). NOM absorbs light in the range of interest and may change the reflectance spectra if care is not taken to remove all excess solution in the film. MIP responses in natural waters compared to LCMS/MS showed in general a good agreement with the gold standard, as shown in the concordance plot, although in some instances, large differences appeared. Discrepancies could be related to particularly challenging conditions in the water matrix, presence of unknown interfering contaminants, or poor reproducibility in the manufacturing process. The use of multiple target sensors has been proposed to identify physicochemical characteristics, leading to a more accurate interpretation of the peak wavelength shift under different types of samples; higher controlled during the synthesis of the films, including automation and larger batches are needed to improve the latter.

Although LCMS/MS is still the gold standard for the analysis of pesticides and its metabolites in natural water samples, portable sensors that can be used in the field offer significant advantages to scientist as a first screening tool for the presence of these compounds in situ. The fabricated sensor showed good potential to be used as a monitoring tool for detection as well as quantification of its targets in natural waters in environmentally relevant concentrations.

## Figures and Tables

**Table 1:** Concentration measurement of ATZ, DEA, and DIA using LCMS/MS and MIP sensors.

Sample Site	ATZ (μg/L) (LCMS)	ATZ (μg/L) (MIP)	DEA (μg/L) (LCMS)	DEA (μg/L) (MIP)	DIA (μg/L) (LCMS)	DIA (μg/L) (MIP)
***12 March 2020***						
T1	0	0	0.19	0.05	0	0.05
T2	0.1	1.77	0.28	0.5	0	1.68
T3	0.56	0.16	1.13	1.41	1.62	1
G1	0.14	0.26	0	0	0	0
G2	0.26	0.32	0.35	0.29	0	0
M	0.6	0.68	1.33	0.84	2.16	1.75
***19 March 2020***						
T1	0	0	0.27	0	0	0.27
T2	0	0	0	0	0	0.33
T3	0.89	0.94	0.77	0.29	1.1	2.12
G1	1.05	0.52	0.52	0.22	0	0
G2	0.32	0.52	0	0.04	0	0
M	0.71	0.17	0.6	0.28	1.03	2.65
L	0.14	0	0	0	0	0
***10 June 2020***						
T1	11.83	12.44	5.11	3.95	2.74	0.91
T2	2.59	1.81	1.4	1.16	1.52	0.63
T3	5.13	4.71	4.01	3.95	3.36	2.21
G1	2.59	1.81	1.88	1.16	1.82	1.85
G2	5.32	5.86	5.1	3	1.73	1.5
M	4.06	4.74	3.88	2.35	5.07	3.9
L	1.79	1.11	1.33	0.58	0	0
***11 August 2020***						
T1	10.71	7.45	10.11	4.55	3.47	2.38
T2	0.22	0.29	0.59	0.22	0.61	0.33
T3	0.42	0.29	0.82	0.93	0.65	0.91
G2	0.51	1.38	0.87	1.16	1.87	2.7
M	0.61	0.72	0.94	1.37	1.68	1.95
L	81.61	25.94	71.59	21.35	12.62	5.95
***23 October 2020***						
T1	2.22	4.71	0.99	2.9	0.84	0.33
T3	0.59	0	0.5	0.5	0.42	1.1
G2	19.67	13.46	3.35	0	1.16	0
M	0.39	0.32	0.7	0	2.07	0.68
***14 March 2021***						
T1	0.97	7.65	1.36	0.54	1.34	0.23
T2	0.16	10.55	0.15	4.62	0.92	0.51
T3	0.2	5.87	0.21	2.36	1.28	3.51
G1	0.15	12.4	0.37	0.15	1.19	0.76
G2	0.2	24.9	0.23	0.5	1.1	0.91
M	0.34	12.61	0.34	0.22	2.29	0
L	3.39	12.4	3.99	1.58	3.35	0.59
***24 April 2021***						
T1	1.39	1.85	1.46	0.63	2.34	2.12
T2	0.33	0.42	0.31	0.63	2.52	2.36
T3	5.72	6.56	0.49	0.22	0.7	0.41
G1	0.54	0.3	0.71	0.35	0	0.32
G2	0.92	0.36	0.51	0	1.26	1.85
M	1.22	0.85	0.77	0.63	1.66	1.62
L	3.09	0.96	3.75	3.94	1.88	0

## Data Availability

The data presented in this study are not available on request from the corresponding author.

## Supplementary Materials

The following supporting information can be downloaded at: https://www.mdpi.com/article/10.3390/molecules27165075/s1, Table S1: Precursor and product ions selected for the analysis of ATR, desethylatrazine (DEA) and deisopropylatrazine (DIA) by HPLC-MS/MS; Table S2: Physicochemical characteristics of samples collected after rain in different seasons; Figure S1: Diverse vegetation in sites T3 (a) and L (b) on the same day (10 June 2020); Figure S2: Effect of salts (NaCl and CaCl_2_) at different ionized strengths on MIPs in absence of atrazine; Figure S3: (a) Comparison of MIPs calibration curve in DI water with MIPs responses in presence of NaCl at 1, 10, and 100 mM levels of ionized strength. (b) MIPs responses in absence and presence of NaCl at different concentration levels and ionic strengths; Figure S4: (a) Comparison of MIPs calibration curve in DI water with MIPs responses in presence of CaCl_2_ at 1, 10, and 100 mM levels of ionized strength. (b) MIPs responses in absence and presence of CaCl_2_ at different concentration levels and ionic strengths; Figure S5: MIPs and NIPs response to atrazine in presence and absence of NOM.
