# Supplementary material for: Detection of Atrazine and Its Metabolites in Natural Water Samples Using Photonic Molecularly Imprinted Sensors
Source: Molecules. 2022 Aug 10;27(16):5075. doi: 10.3390/molecules27165075 (PMC9415651; doi:10.3390/molecules27165075)
Supplement: Supplementary file 1 [file molecules-27-05075-s001.zip › molecules-1832283-supplementary.pdf]

Detection of atrazine and its metabolites in natural water samples using photonic molecularly imprinted sensors

Zahra Salahshoor [1], Khanh-Van Ho [2,3,4,5], Shu-Yu Hsu [2] , Adel H. Hossain[1], Kathleen Trauth [1], Chung-Ho Lin [2] and Maria Fidalgo [1,*]

[1] Department of Civil and Environmental Engineering, University of Missouri, Columbia MO; zsq73@umsystem.edu; ahhd49@umsystem.edu; trauthk@missouri.edu; fidalgom@missouri.edu

[2] Center for Agroforestry, School of Natural Resources, University of Missouri, Columbia MO; vkh6c6@missouri.edu; shgm5@mail.missouri.edu linchu@missouri.edu

3 Department of Chemistry, University of Missouri, Columbia, MO 65211, USA

4 Molecular Imaging and Theranostics Center, University of Missouri, Columbia, MO 65211, USA

5 Department of Food Technology, Can Tho University, Can Tho, Vietnam.

* Correspondence: fidalgom@missouri.edu

Table S1. Precursor and product ions selected for the analysis of ATR, desethylatrazine (DEA) and deisopropylatrazine (DIA) by HPLC-MS/MS (LOD = limit of detection).

Compound	RT (min)	Molecular Ions (m/z)	Product Ions (m/z)	Polarity	Linear equation	Correlation Coefficient (R square)	Collision energy	Cone Voltage (V)	LOD (ug/L)
ATR	9.471	215.86	173.89	ES+	y=58890x	0.9986	Tune	30	0.06
DEA	7.632	187.86	145.78	ES+	y=10336x	0.9957	Tune	30	1.45
DIA	6.829	173.82	131.76	ES+	y=3321x	0.9973	Tune	30	5.72

Table S2. Physicochemical characteristics of samples collected after rain in different seasons.(pH and temperature were measured on-site, TOC and conductivity were measured in the laboratory).

Sample site	TOC (mg/L)	Conductivity (µmhos/cm)	pH	Temperature (°C)
March 12th,2020				
T1	3.155	92.2	7.25	10.2
T2	~0	173.8	7.79	11.1
T3	2.121	91	7.3	11
G1	4.287	144.9	7.45	11
G2	2.052	94.8	7.45	11.1
M	3.572	120.5	7.32	11.15
L				
March 19th, 2020				
T1	2.026	42.9	7.49	17
T2	4.265	59.2	7.61	18.2
T3	4.104	33	7.21	17.7

G1	1.617	46.8	7.3	17.9
G2	2.548	61.5	7.58	17.7
M	4.437	40.8	7.01	18.7
L	~0	74.1	7.22	18.7
June 10th, 2020				
T1	9.957	52.4	7.92	21.5
T2	8.592	96.5	7.52	21.2
T3	8.501	63.7	7.31	21.1
G1	4.216	108.5	7.4	21.4
G2	10.80	85.7	7.36	22
M	7.280	99	6.93	21.8
L	2.016	134.1	7.35	23.4
August 11th, 2020				
T1	3.036	48.2	7.97	26.8
T2	7.196	116.1	7.57	27.4
T3	3.270	57.1	7.39	28.5
G1	10.93	116.5	7.4	30
G2	7.304	95.2	7.63	31.6
M	2.212	63.5	7.13	29.7
L	2.422	132.5	7.04	30.4
October 23rd, 2020				
T1	~0	171	8.13	9.8
T2				
T3	~0	177.4	7.53	9.7
G1				
G2	5.618	105.8	7.34	10.8
M	7.002	111	7.79	10.6
L				
March 14th, 2021				
T1	1.684	109	8.14	13
T2	1.589	166.5	7.9	11.4
T3	3.493	169.6	7.52	8.7
G1	3.534	149.2	7.61	8.3
G2	2.782	104.3	7.47	7.9
M	2.479	197	7.65	7.7
L	0.618	167.4	7.32	7.2
April 24th, 2021				
T1	0.050	97.7	7.93	20.2
T2	6.193	113.8	7.64	21.9
T3	0.320	101	7.59	20.9
G1	8.030	84.5	7.65	20.6
G2	1.041	90	7.48	19.8
M	~0	108.6	7.77	18.6
L	3.153	124.4	7.45	19.6

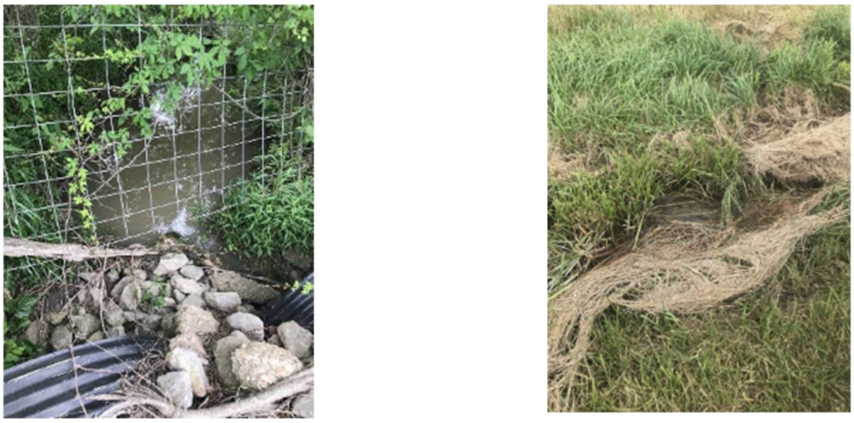

(**a**) (**b**)

Figure S1. Diverse vegetation in sites T3 (a) and L (b) on the same day (Jun. 10th, 2020)

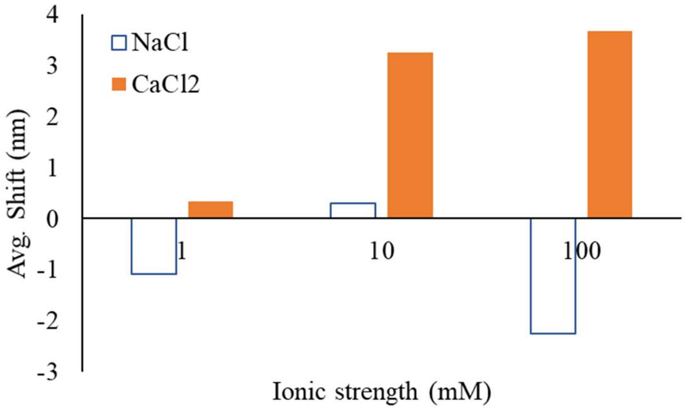

Figure S2. Effect of salts (NaCl and $CaCl_2$) at different ionized strengths on MIPs in absence of atrazine.

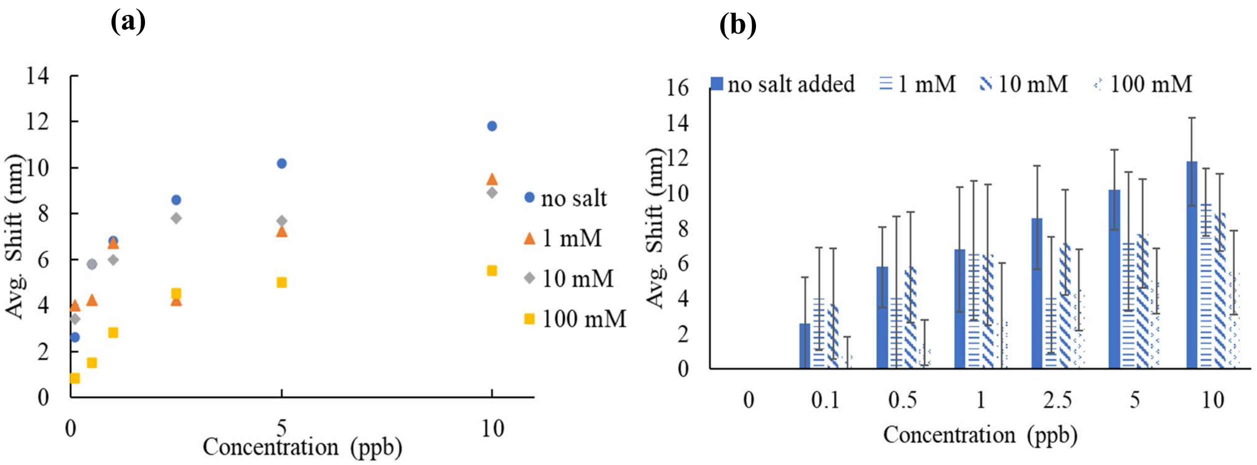

Figure S3. a) Comparison of MIPs calibration curve in DI water with MIPs responses in presence of NaCl at 1, 10, and 100 mM levels of ionized strength. b) MIPs responses in absence and presence of NaCl at different concentration levels and ionic strengths.

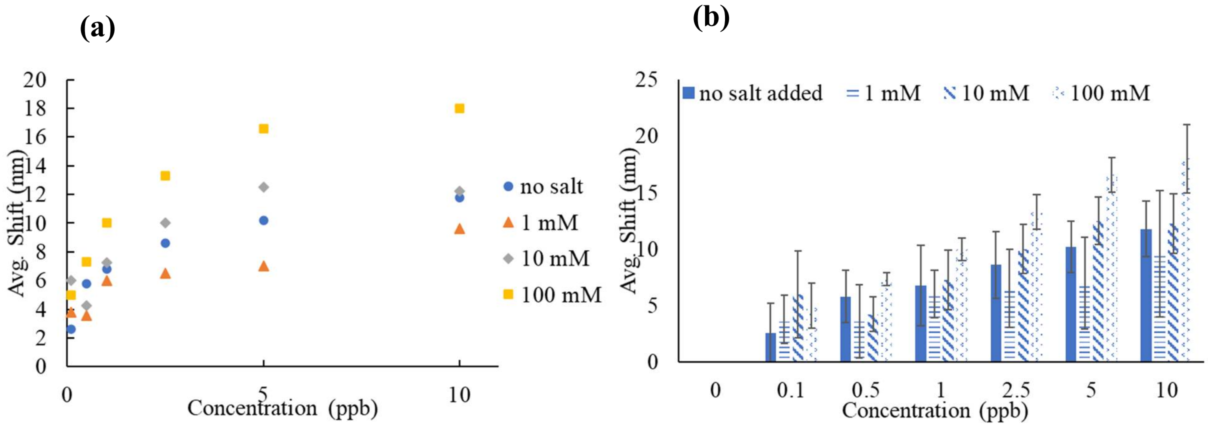

Figure S4. a) Comparison of MIPs calibration curve in DI water with MIPs responses in presence of $CaCl_2$ at 1, 10, and 100 mM levels of ionized strength. b) MIPs responses in absence and presence of $CaCl_2$ at different concentration levels and ionic strengths.

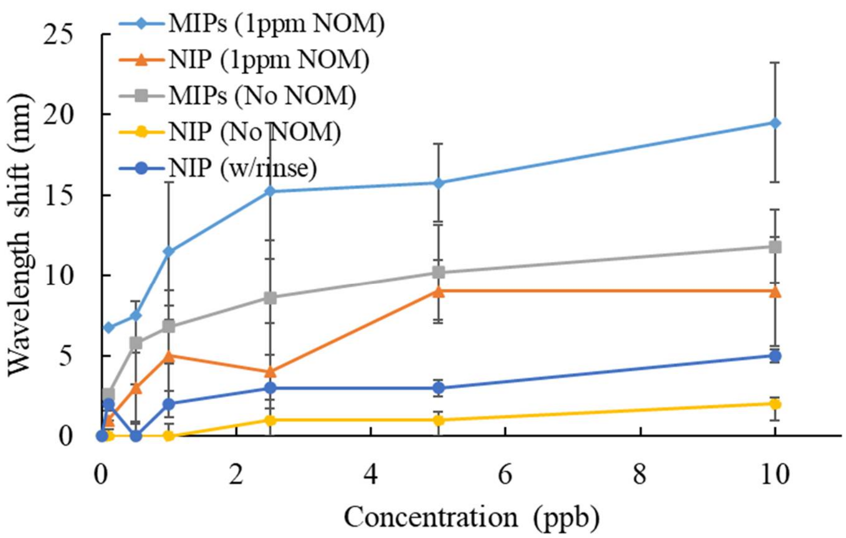

Figure S5. MIPs and NIPs response to atrazine in presence and absence of NOM.
